# Odontological Society of Pennsylvania

**Published:** 1889-02

**Authors:** I. G. Baumgardner


### Reports of Society Meetings.

### THE ODONTOLOGICAL SOCIETY OF PENNSYLVANIA.

Especially Reported for the INTERNATIONAL by I. G. BAUMGARDNER, D. D. S.

The regular meeting of the Odontological Society of Pennsylvania was held Saturday evening, December 1, 1888, at Justi's Rooms, Arch and Thirteenth streets. President KIRK in the Chair.

### INCIDENTS OF PRACTICE.

*Dr. C. N. Pierce*—About three weeks ago I implanted the left superior lateral incisor, and although it is early to predict results yet, to-day it has the appearance of being in every way satisfactory. There is nothing especially worthy of note in the case, save that the patient had been wearing an artificial lateral attached to the cuspid, with its base resting upon the gum without protection. The constant pressure had caused absorption, not only of the gum, but also of the labial process, so that the tissue was quite deficient. An incision was made in the gum so as to protect it as much as possible. Then, with some drills obtained through the kindness of Dr. Kirk, a hole of suitable size to receive the root of the selected tooth was made, and the tooth inserted and retained without ligatures, the slight twist or curve in the root being sufficient to hold it in place.

In drilling the process a small piece of the apical end of the original root was found, but in no way interfered with the progress of the operation nor with its ultimate success, judging from its present appearance, which is only slightly marred by a deficiency of gum on the labial surface.

I would like to ask, are we not every year more indebted to antiseptics? The use of mouth-washes preserve the teeth and prevent the progress of decay. During the last two or three years I have had very good results from antiseptic washes, and I am satisfied that in persons between eight and twenty and in adult life, where there is a tendency, it modifies the progress of caries, and will protect our work and protect us. Years ago I used to speak slightingly of mouth-washes. I thought powders were better than washes, but am convinced that if properly prepared, free from foreign substances, they are an advantage in the preservation of the teeth.

*Dr. Jefferis.*—A case presented itself to me a few weeks ago—a wisdom tooth, that from its position could not erupt. It was in the mouth of a gentleman about 30 years of age, who had suffered quite a while with neuralgia. He had splendid, large teeth, and had, at the advice of a dentist, his left superior wisdom tooth extracted, from the supposition that crowded teeth caused the neuralgia. This, however, afforded no relief. He fell into my hands about three weeks ago, and upon examining the place where the tooth was I found a single root, which I removed, and found it to be the root of a wisdom tooth. In searching further with an excavator, I observed by sounding away up on a line with the roots of the other molar teeth what appeared to be a crown of a tooth. I extracted the second molar and found a fully developed wisdom tooth, with a long root, which was the cause of the neuralgia, and which I also extracted. It was a case of a second wisdom tooth in the same place.

*Dr. H. C. Register*—As it is desirable to keep a record of the implantation cases, I report one performed upon Prof. P., of Jefferson Medical College, this city. He had been using an artificial crown upon the R. S. lateral for several years, but was unforunate enough, while in Europe this past summer, to fracture the root longitudinally; and as the crown was very short, the teeth striking directly upon their cutting edges, I deemed it best to try implantation in preference to using plate or bridge, and extracted the root and inserted a natural tooth at the same sitting. The operation differed a little from the ordinary implantation operations, as there was a natural socket already formed, but not of sufficient depth to receive the tooth; and I deepened it, and after disinfecting and filling canal of tooth, forced it into position under pressure of a wood cushion forcibly malletted into position. There was little or no mobility about the tooth after its insertion. The operation was followed with little soreness, which disappeared after the third or fourth day, except when pressure was made. There was a little mobility during these several days of soreness, but no disposition for the tooth to elongate. After the seventh or eighth day, in response to tapping, there was no soreness, and the sound indicating direct union with the bony tissue. The gum festoon lies as perfectly as upon any of the other teeth. There was no issue of pus following the operation, and after the fourteenth or fifteenth day the patient used it as he would any of his other teeth.

I wrote to Professor P. in regard to his case, and received the following reply:

"I have not the date of the 'planting,' but for nearly a month after I was occasionally painfully reminded of the new tooth.

However, by the end of the month it seemed firm, and gradually all sensitiveness upon moderate pressure disappeared. The tooth now is almost as useful as its predecessor, even in its best days; only a little discomfort when very firm pressure is made in eating; but this discomfort is only trivial and temporary."

*Dr. Kirk.*—Whatever may be said of tobacco on moral grounds, there is certainly a retarding of decay in the teeth of those who use tobacco, whether it be smoked or chewed. I draw attention to it because it is an instance of an antiseptic material in the mouth giving indisputable results in the arrest and retarding of caries. If one antiseptic will do it, why not another?

*Dr. James Truman.*—It seems to me that Prof. Pierce has touched an important question. Antiseptics will, without doubt, occupy a leading position in the therapy of the future. This is true of dentistry at the present time. The large number of micro-organic forms found in the oval cavity lead to the inference that disease, both general and special, should be met and combatted. Pathogenic forms should be destroyed before they have time to enter the general circulation. Very little attention is paid to this in general practice. Antiseptics will be freely used in sick rooms, but the mouths of those exposed to contagious diseases are probably never looked after. The time is certainly coming when the condition of the mouth will be examined by the general practitioner with more care than he does now the sanitary state of the sick room. My view is that if an antiseptic be used regularly two or three times a day, and especially at night before retiring, there would be but little to fear from contagion through the atmosphere. It is not, I think, necessary that a germicide be used, the inhibiting property of many agents may be quite sufficient, and if used in the form of spray, as recommended by Dr. H. C. Register, the effect must be better, not only in the mouth, but in the air-passages, where it can be applied effectively.

*Dr. Register.*—Dr. Truman kindly referred to the spray apparatus used in my practice. It is really wonderful to note the benefits to those who use an antiseptic through an atomizer. When patients come to me semi-annually or annually I notice that in mouths that with the greatest difficulty were kept in good condition formerly, and in which is now used the atomizer, there is a very decided improvement. The word decided hardly expresses it. The mouth is clean, the surfaces of the teeth are clean. The places (boundary lines) that are protected by fillings are cleaner than ever I have known them to be since I commenced practice. Among simple anæsthetics recently recommended is oil of peppermint—children are very fond of it, tasting like candy, and these little people, as well as older patients, have no difficulty in forming habits of using the atomizer and washing away these gummy substances that cling in the interstices of teeth, and which, in my judgment, are the principal cause of caries, being the medium in which bacteria reside and luxuriantly grow, while feeding upon the oral environment and decalcified tooth matter, acidulating and degenerate organisms being coincident.

*Dr. C. N. Pierce*—I would like to ask Dr. Sudduth whether in his investigations he has found an antiseptic that is best for all cases?

*Dr. Sudduth*—While I have given considerable attention to the subject, I am not ready to report. Of the three classes of antiseptics required in the mouth, I am satisfied that bichloride of mercury is the best we can use in root canals, where no deleterious results can arise from its use. Then for a stimulant antiseptic, we have carbolic acid. I have not yet decided upon one for daily use in the mouth. Hydronapthol has been recommended, also silica-fluoride of soda with its different combinations of zinc and bismuth. My investigations have not progressed sufficiently to unqualifiedly recommend any of these; silica-fluoride of soda inhibits the development of moulds and fungi in general. Oil of peppermint, wintergreen and cinnamon make good antiseptics and are not objectionable when used in the mouth.

*Dr. Kirk*—Listerine is a very agreeable antiseptic mouth wash.

